# The Cell Envelope Integrity Protein Homologue D0Y85_RS06240 of *Stenotrophomonas* Confers Multiantibiotic Resistance

**DOI:** 10.1155/2024/7547514

**Published:** 2024-01-20

**Authors:** Chuanqing Zhong, Xiaoqiang Deng, Aihua Jiang, Yayu Liu, Yuanyuan Liu, Jiafang Fu, Guangxiang Cao

**Affiliations:** ^1^School of Municipal and Environmental Engineering, Shandong Jianzhu University, Ji'nan 250101, China; ^2^Jinan Urban and Rural Water Bureau, Ji'nan 250099, China; ^3^Biomedical Sciences College, Shandong Medicinal Biotechnology Centre, Shandong First Medical University and Shandong Academy of Medical Sciences, Ji'nan 250117, China

## Abstract

**Background:**

The potential role of cell envelope integrity proteins in mediating antibiotic resistance is not well understood. In this study, we investigated whether the cell envelope integrity protein D0Y85_RS06240 from the multiantibiotic resistant strain *Stenotrophomonas* sp. G4 mediates antibiotic resistance.

**Methods:**

Bioinformatics analysis was conducted to identify proteins related to the D0Y85_RS06240 protein. The D0Y85_RS06240 gene was heterologously expressed in *Escherichia coli*, both antibiotic MICs and the effect of efflux pump inhibitors on antibiotic MICs were determined by the broth microdilution method. A combination of antibiotic and efflux pump inhibitor was used to investigate bacterial killing kinetics, and binding of D0Y85_RS06240 to antibiotic molecules was predicted by molecular docking analysis.

**Results:**

Sequence homology analysis revealed that D0Y85_RS06240 was related to cell envelope integrity proteins. The D0Y85_RS06240 heterologous expression strains were resistant to multiple antibiotics, including colistin, tetracycline, and cefixime. However, the efflux pump inhibitor N-methylpyrrolidone (NMP) reduced the antibiotic MICs of the D0Y85_RS06240 heterologous expression strain, and bacterial killing kinetics revealed that NMP enhanced the bactericidal rate of tetracycline to the drug-resistant bacteria. Molecular docking analysis indicated that D0Y85_RS06240 could bind colistin, tetracycline, and cefixime.

**Conclusion:**

The cell envelope integrity protein D0Y85_RS06240 in *Stenotrophomonas* sp. G4 mediates multiantibiotic resistance. This study lays the foundation for an in-depth analysis of D0Y85_RS06240-mediated antibiotic resistance mechanisms and the use of D0Y85_RS06240 as a target for the treatment of multiantibiotic-resistant bacterial infections.

## 1. Introduction

The long-term overuse of antibiotics in the aquaculture and medical fields has led to increasing concentrations of antibiotics in the environment [1], and evolutionary pressure from antibiotic exposure can select for bacterial antibiotic resistance, reducing the therapeutic effect of antibiotics [2]. Bacteria develop resistance to antibiotics mainly through enzymatic degradation, efflux pumps, and target site modification, mechanisms that can enable resistance to one or more antibiotics [3]. Antibiotic-resistant bacteria and the corresponding antibiotic resistance genes are considered new environmental pollutants and pose great risks in the environment [4–6]. For example, sulfonamide- and tetracycline-resistant opportunistic bacteria (e.g., *Acinetobacter* spp.) and the corresponding antibiotic resistance genes (e.g., *tetM*, *tetO*, *tetT*, *tetW*, *sul1*, and *sul2*) were enriched in an aquaculture environment [7]. The increasing number of antibiotic-resistant bacteria that pollute soil and water environments, and particularly the increasing number of “superbugs,” is a direct or indirect threat to human health [8].

Efflux pumps have been reported to mediate multiple antibiotic resistances in bacteria and are capable of transporting antibiotics out of the bacterial cell. Efflux pumps lower intracellular antibiotic concentrations, allowing bacteria to survive under greater concentrations of antibiotics [9]. Studies have shown that some membrane integrins also act as efflux pumps that mediate bacterial tolerance to various compounds including antibiotics. Mascio et al. found that the integral membrane protein ArsB is involved in bacterial arsenite and antimonate resistance [10], and members of the DedA family of membrane integrins, which are widespread in bacteria, are associated with resistance to multiple antibiotics [11]. However, the characteristics and specific functions of cell envelope integrity proteins need to be further studied.

The bacterial species *Stenotrophomonas maltophilia* is an opportunistic pathogen that can cause nosocomial infections, and its increasingly broad antibiotic resistance can seriously impact the health of patients [12]. *Stenotrophomonas* sp. G4 is a multiple antibiotic-resistant strain, which is phylogenetically distinct from *S*. *maltophilia* [13]. Genome annotation identified the *Stenotrophomonas* sp. G4 gene D0Y85_RS06240 as a cell envelope integrity protein, and our goals in this study were to investigate whether D0Y85_RS06240 might contribute to the antibiotic resistance of this strain. Two heterologous expression strains containing D0Y85_RS06240 were constructed to analyze the effect of D0Y85_RS06240 on antibiotic resistance and to gain further insights into the mechanisms of antibiotic resistance in bacteria.

## 2. Materials and Methods

### 2.1. Strains and Vectors

The *Stenotrophomonas* sp. G4 strain was isolated from sewage water by our research group and maintained in our laboratory [13]. *Escherichia coli* DH5*α* was purchased from Shanghai Angyu Biotechnology Co., and the pMD18-T vector was purchased from Beijing Takara Company. *E. coli* strain DR06240 and strain DP06240 were constructed in this study.

### 2.2. Primers

The primers used in this study were synthesized by Biological Bioengineering (Shanghai), and the primer sequences are shown in Table 1.

### 2.3. Construction of Strain DR06240 Expressing D0Y85_RS06240 from Its Native Promoter

Gene D0Y85_RS06240 along with its 148-bp native promoter was amplified by PCR with primer pair RS06240-F/RS06240-R (Table 1), using genomic DNA of *Stenotrophomonas* sp. G4 as a template. The PCR products were recovered and ligated with the vector pMD18-T to generate a recombinant vector pMD-RS06240, which was transformed into *E. coli* DH5*α*. Transformants were screened, and the selected transformant was named strain DR06240. The presence of pMD-RS06240 in DR06240 was confirmed by extraction of the recombinant vector followed by restriction enzyme digestion and DNA sequencing.

### 2.4. Construction of Strain DP06240 Expressing D0Y85_RS06240 from a Constitutive *E. coli* Promoter

The reading frame sequence of the D0Y85_RS06240 gene was amplified by PCR with primer pair P06240-F/P06240-R (Table 1) and G4 genomic DNA as a template. At the same time, a constitutive promoter DNA fragment of *E. coli* was amplified with a primer pair AP-F/AP-R (Table 1) and the pMD18-T vector as template. The above PCR products were then mixed to serve as PCR template, and PCR overlapping was used to amplify the fusion fragment of the *E. coli* promoter and the D0Y85_RS06240 gene with primer pair AP-F and P06240-R. Then, the PCR product was recovered, ligated with the pMD18-T vector to generate the recombinant vector pMD-P06240, and then pMD-P06240 was transferred into *E. coli* DH5*α*, followed by screening of transformants, with the selected transformant named DP06240. The presence of pMD-P06240 in DP06240 was confirmed by extraction of the recombinant vector followed by restriction enzyme digestion and DNA sequencing.

### 2.5. Measurement of Antibiotic MICs by the Broth Microdilution Method

The antibiotic MICs of strain DR06240 and DP06240 were determined using the broth microdilution method [14]. Cultures in the log phase of growth (OD_600_ = 0.6) were diluted to 1 × 10^6^ to 2 × 10^6^ colony-forming units/mL (CFU/mL), and then 100 *μ*L of LB liquid medium and 100 *μ*L diluted were added per well in a 96-well plate with or without antibiotics. Then, the 96-well plate was placed in a 37°C incubator for 16–24 h. The OD_600_ values were determined by microplate absorbance reader (HBS-1096C, Detie) using strain DH5*α* carrying the pMD18-T vector as control. Five replicates of each sample were set up for each treatment.

### 2.6. Analysis of the Effects of Efflux Pump Inhibitors on Antibiotic MICs

The effects of efflux pump inhibitors on the antibiotic MICs of the test strains were also analyzed using the broth microdilution method [14]. The selected efflux pump inhibitors were verapamil (VER), reserpine (RES), carbonyl cyanide 3-chlorophenylhydrazone (CCCP), and N-methylpyrrolidone (NMP) [15]. First, the effects of different concentrations of VER, RES, CCCP, and NMP on the growth of the test strains were determined by the broth microdilution method in order to identify the maximum concentration that did not inhibit strain growth. Then, the antibiotic MICs were determined in the presence of the identified concentrations of the efflux pump inhibitors.

### 2.7. Bacterial Killing Kinetics Assay

The bacterial killing kinetics assay was carried out as previously described with some modifications [16, 17]. Strain DP06240 was cultured in LB broth to exponential phase and diluted to 10^6^ CFU/mL with fresh LB broth. Then, 32 mg/L tetracycline (TET group), 8 mg/L NMP (NMP group), or 32 mg/L tetracycline and 8 mg/L NMP (COM group) were added to the bacterial suspensions followed by incubation at 37°C for 0, 30, 60, 90, 120, and 180 min. Untreated bacteria were used as the control group. At each time point, 100 *μ*L bacterial cultures were taken and diluted 1000-fold with fresh LB broth, and 100 *μ*L of the dilution was spread on LB solid medium. The plates were incubated at 37°C overnight, and the colonies were counted.

### 2.8. D0Y85_RS06240 Sequence Alignment Analysis

The NCBI Prokaryotic Genome Annotation Pipeline (PGAP) program was used to obtain the functional information and amino acid sequence of the protein encoded by the D0Y85_RS06240 gene. Gene annotation was also performed using the RASTtk server [18]. The NCBI-BLASTp online program was applied to find homologous proteins of D0Y85_RS06240 (https://blast.ncbi.nlm.nih.gov/Blast.cgi), and the amino acid sequences of the homologous proteins were input into the CLUSTALW software for comparison. The ESPript3 program was used to view the alignment results [19].

### 2.9. Molecular Docking Analysis of the D0Y85_RS06240 Protein and Antibiotics

The homology modeling of D0Y85_RS06240 protein was performed using SWISS-MODEL online software (https://swissmodel.expasy.org/) to predict the 3D model of D0Y85_RS06240 protein, and the small molecule ligand website (https://www.chemspider.com/) was used to find the molecular structures of the selected antibiotics for analysis. Then, the 3D model of protein D0Y85_RS06240 and the molecular structure of each antibiotic were imported into Discovery Studio 2.0 software, and the docking analysis was performed by applying the precise molecular docking technique (CDOCKER) [20].

## 3. Results

### 3.1. Amino Acid Sequence Analysis of D0Y85_RS06240 and Homologous Proteins

**Figure 1:**
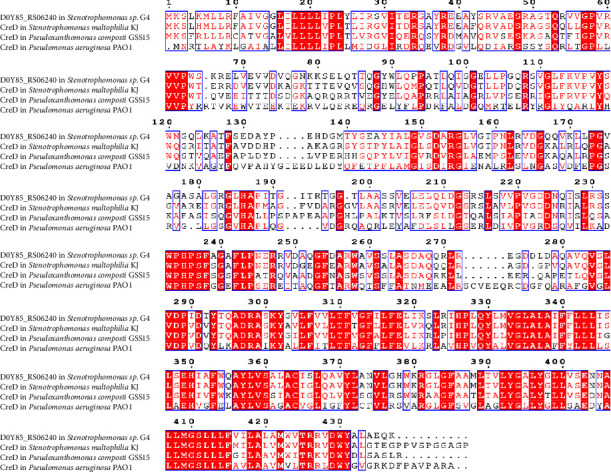
Homology analysis of amino acid sequences of D0Y85_RS06240 and related proteins. Red shading indicates identical residues and red font indicates similar amino acids.

Genome annotation indicated that D0Y85_RS06240 encodes a cell envelope integrity protein in the *Stenotrophomonas* sp. G4 strain. Comparative analysis showed that the amino acid similarity of D0Y85_RS06240 with the CreD cell envelope integrity proteins from *S. maltophilia* KJ, *Pseudoxanthomonas composti* GSS15, and *Pseudoxanthomonas aeruginosa* PAO1 was 76.4%, 57.7%, and 42.5%, respectively (Figure 1). Notably, CreD in *P. aeruginosa* PAO1 was found to be associated with *β*-lactam antibiotic resistance [21].

### 3.2. Heterologous Expression of D0Y85_RS06240 in *E. coli*

**Figure 2:**
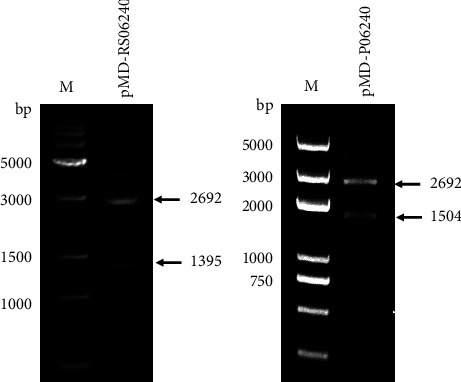
Confirmation of the presence of D0Y85_RS06240 expression vectors in recombinant strains. (a) pMD-RS06240 and (b) pMD-P06240 were extracted, respectively, from strains DR06240 and DP06240 and then digested with *Hind* III and *Xba* I, followed by electrophoresis on agarose gels. M in (a), 15K DNA marker (BM161-01, trans, China). M in (b), DL5000 DNA marker (BDIT0040, rainbio, China).

In this study, we constructed *E. coli* strains DR06240 and DP06240, which contain gene D0Y85_RS06240 controlled by its native promoter or by a constitutive *E. coli* promoter, respectively. The presence of the recombinant vector pMD-RS06240 in strain DR06240 and pMD-P06240 in strain DP06240 was verified as shown in Figure 2 and were further confirmed by DNA sequencing. Hence, both the restriction enzyme digestion and DNA sequencing confirmed the successful construction of the recombinant strains DR06240 and DP06240.

### 3.3. Antibiotic MICs of the Recombinant Strains

The antibiotic MICs of the DR06240 and DP06240 strains were determined by the microdilution method, using the DH5*α* (18T) strain carrying the pMD18-T vector as a control (Table 2). Results showed that strain DR06240 had increased resistance to colistin and tetracycline, as the MIC values were 4 mg/L and 16 mg/L, respectively, which were higher than the MICs for the control strain DH5*α* (18T). However, the MICs of colistin, cefixime, and tetracycline for the strain DP06240 were ≥128 mg/L, 32 mg/L, and ≥128 mg/L, respectively, which were markedly higher than those of DH5*α* (18T) and DR06240.

### 3.4. Efflux Pump Inhibitor NMP Inhibits the Function of D0Y85_RS06240

Antibiotic MIC values indicated that the DP06240 strain was markedly resistant to colistin, tetracycline, and cefixime. To test whether the D0Y85_RS06240 protein acts as an efflux pump, we examined the effects of the known efflux pump inhibitors VER, RES, CCCP, and NMP on the antibiotic MICs of DP06240. For this assay, we selected concentrations of the inhibitors that had no significant effect on the growth of strain DP06240, specifically, 8 mg/L VER, 8 mg/L RES, 0.1 mg/L CCCP, and 8 mg/L NMP. Results showed that the proton-pump inhibitors VER, RES, and CCCP had no effect on the antibiotic MICs of strain DP06240 (Table 3). In contrast, the inhibitor NMP markedly reduced the colistin and tetracycline MICs of strain DP06240, lowering the colistin MIC value from ≥128 mg/L to 64 mg/L, the cefixime MIC value from 32 mg/L to 16 mg/L, and the tetracycline MIC value from ≥128 mg/L to 16 mg/L (Table 3).

### 3.5. Bactericidal Effect of Tetracycline Combined with NMP on Strain DP06240

To test the antimicrobial effect of tetracycline combined with NMP, a bacterial killing kinetics assay was performed using a colony-counting method. Results indicated that tetracycline combined with NMP showed rapid killing kinetics toward strain DP06240, with declines in CFU observable following 30 to 60 min of exposure when compared to tetracycline alone (Table 4). Even more notably, the CFU level dropped to zero when the incubation time was extended to 180 min, which implied that the antimicrobial effect of the tetracycline/NMP combination was bactericidal rather than bacteriostatic. In contrast, tetracycline alone could not completely eliminate *E. coli* DP06240.

### 3.6. Molecular Docking of the D0Y85_RS06240 Protein with Antibiotics

**Figure 3:**
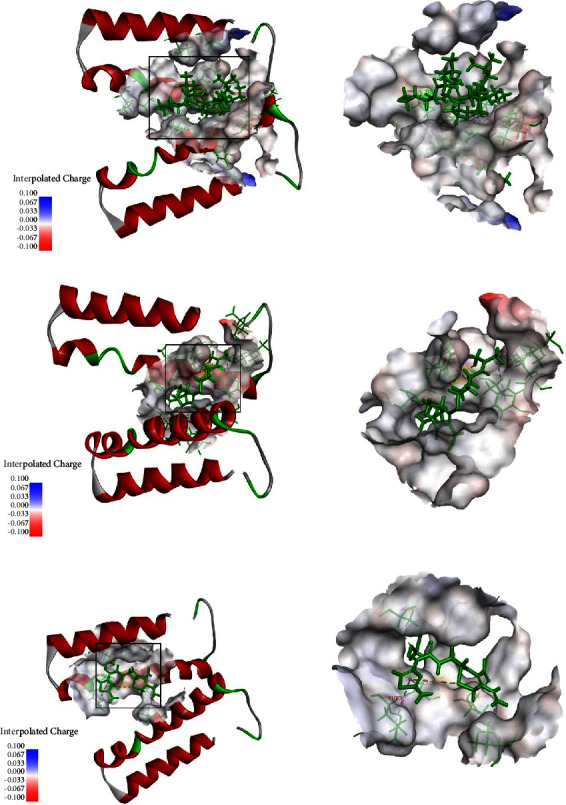
Molecular docking analysis of D0Y85_RS06240 with antibiotics. Molecular docking diagrams of D0Y85_RS06240 with (a) colistin, (b) cefixime, and (c) tetracycline.

A homology model of D0Y85_RS06240 was constructed based on the crystal structure of 5khs s.1, an efflux transporter from *Burkholderia multivorans*. Molecular docking analysis showed that the pore structure formed by a D0Y85_RS06240 dimer could bind to colistin, cefixime, and tetracycline (Figure 3), but not bind to florfenicol, meropenem, ciprofloxacin, or kanamycin, which was consistent with the antibiotic MICs for the D0Y85_RS06240 recombinant strains. Binding site analysis showed that colistin binds to amino acid residues Thr390, Ala395, Tyr397, Gly398, and Leu399 of the D0Y85_RS06240 protein (Figure 3(a)); cefixime binds to amino acid residues Ala369, Leu383, Ala386, Thr390, and Val391 (Figure 3(b)); and tetracycline binds to amino acid residues Thr390, Val391, Gly394, and Ala395 (Figure 3(c)). In addition, molecular docking analysis showed that colistin, cefixime, and tetracycline all bind to the C-terminal amino acid residues of the dimeric D0Y85_RS06240 protein.

## 4. Discussion

Amino acid sequence analysis revealed that the cell envelope integrity protein D0Y85_RS06240 from *Stenotrophomonas* sp. G4 is highly homologous to the cell envelope integrity protein CreD from *S. maltophilia*, *P. composti*, and *P. aeruginosa*. CreD of *P. aeruginosa* PAO1 was reported to be associated with *β*-lactam antibiotic resistance [21], whereas CreD of *S. maltophilia* KJ was found not to be associated with *β*-lactam antibiotic resistance [22].

In this study, we explored whether D0Y85_RS06240 from *Stenotrophomonas* sp. G4 mediates antibiotic resistance. Recombinant heterologous expression strains expressing D0Y85_RS06240 from either its native promoter (strain DR06240) or an *E. coli* promoter (strain DP06240) were constructed using *E. coli*. The MICs of colistin, tetracycline, and cefixime were higher in DR06240 and DP06240 than in the control strain DH5*α* (18T), while the florfenicol, meropenem, ciprofloxacin, and kanamycin MICs were the same in DR06240, DP06240, and DH5*α* (18T), indicating that heterologous expression of D0Y85_RS06240 only mediated resistance to colistin, tetracycline, and cefixime. Moreover, the antibiotic MICs of DP06240 were notably higher than those of DR06240, indicating that the expression level of the D0Y85_RS06240 gene might be higher in DP06240 than in DR06240. The transcription of the D0Y85_RS06240 gene in DP06240 is controlled by a constitutive *E. coli* promoter, which may result in higher expression of D0Y85_RS06240 and therefore, higher resistance of DP06240 to antibiotics, suggesting that increased expression of D0Y85_RS06240 could enhance bacterial resistance to antibiotics. The differences in gene expression may be related to where genes are located in the microenvironment of the host bacteria, or to the promoter [23, 24], with the combination of the *E. coli* host promoter and an *E. coli* strain enabling enhanced expression of D0Y85_RS06240.

MIC data showed that the proton pump inhibitors VER, RES, and CCCP had no effect on the antibiotic MICs of strain DP06240, suggesting that D0Y85_RS06240 is not a proton-driven efflux pump. NMP, a known efflux pump inhibitor, has been shown to reverse multidrug resistance in *E. coli* overexpressing efflux pumps [25], and we found that NMP markedly reduced the MICs of colistin, cefixime, and tetracycline for *E. coli* DP06240, meanwhile the growth of strain DP06240 was not affected when the tested concentration of 8 mg/L NMP was added alone, indicating that NMP was able to inhibit the function of D0Y85_RS06240 and further suggesting that the cell envelope integrity protein D0Y85_RS06240 may function as an efflux pump. In addition, the more rapid bactericidal action of the tetracycline/NMP combination provides impetus for screening for novel D0Y85_RS06240 inhibitors and for using D0Y85_RS06240 as a target to treat antibiotic-resistant bacteria.

## 5. Conclusion

Gene annotation and homologous protein alignment identified D0Y85_RS06240 of the *Stenotrophomonas* sp. G4 strain as a cell envelope integrity protein. MIC assays showed that heterologous expression of D0Y85_RS06240 conferred host resistance to colistin, cefixime, and tetracycline, and that the efflux pump inhibitor NMP could reduce the antibiotic MICs of the host strains. Additionally, a bacterial killing kinetics assay revealed that NMP could enhance the bactericidal rate of tetracycline on drug-resistant bacteria, suggesting that inhibition of D0Y85_RS06240 can be enhanced with concomitant treatment of strain DP06240 by tetracycline and NMP. Molecular docking analysis further showed that D0Y85_RS06240 can bind to colistin, tetracycline, and cefixime, consistent with the ability of D0Y85_RS06240 to confer resistance to these three antibiotics. Our findings lay the foundation for the in-depth analysis of the resistance mechanism of the membrane integrin protein D0Y85_RS06240 and the potential for using D0Y85_RS06240 as a target for the treatment of drug-resistant bacterial infections.

## Figures and Tables

**Table 1:** Primers used in this study.

Primer	DNA sequence (5′⟶3′)
RS06240-F	TCGGGGCTGGCTTAAAGCACGAAGAACGGGCTGTC
RS06240-R	CACAGGAAACAGCTAATCGCCAGCGCTACTGCCAG
P06240-F	AATATTGAAAAAGGAAGAGTATGAAATCCCTGAAGATGCT
P06240-R	TTACTTCTGTTCGGCCAGGG
AP-F	GCCTCGTGATACGCCTATTT
AP-R	ACTCTTCCTTTTTCAATATT

**Table 2:** Antibiotic MICs of DR06240 and DP06240.

Antibiotic	DR06240	DP06240	DH5*α* (18T)
Colistin	4^*R*^*∗*^^	≥128^R^	<2^S^
Cefixime	2^S^	32^R^	<2^S^
Meropenem	<2^S^	<2^S^	<2^S^
Florfenicol	4^S^	4^S^	4^S^
Ciprofloxacin	2^S^	2^S^	2^S^
Kanamycin	4^S^	4^S^	4^S^
Tetracycline	16^R^	≥128^R^	2^S^

^*∗*^MIC in mg/L; ^R^resistant; ^S^susceptible. MIC breakpoint was based on CLSI guidelines. DH5*α* (18T) is the control strain.

**Table 3:** Effects of efflux pump inhibitors on MICs of strain DP06240.

Inhibitors	Colistin	Cefixime	Tetracycline
VER	≥128^*∗*^	32	≥128
RES	≥128	32	≥128
CCCP	≥128	32	≥128
NMP	64	16	16
No inhibitor	≥128	32	≥128

^*∗*^MIC in mg/L.

**Table 4:** Bactericidal effects of the combination of tetracycline and NMP on strain DP06240.

Time (min)	Colony-forming units/mL (×10^6^)			
	Control	TET^*∗*^	NMP	COM
0	203 ± 18	196 ± 13	209 ± 15	201 ± 17
30	256 ± 21	133 ± 22	214 ± 18	45 ± 4.23
60	360 ± 40	96 ± 3.42	293 ± 21	15.11 ± 0.12
90	450 ± 39	60.34 ± 2.14	384 ± 11	6.20 ± 0.31
120	502 ± 63	40.15 ± 1.23	411 ± 32	1.61 ± 0.05
180	623 ± 70	45.14 ± 0.32	464 ± 27	0 ± 0.00

^*∗*^TET, tetracycline; NMP, N-methylpyrrolidone; COM, tetracycline and NMP combination.

## Data Availability

No underlying data were collected or produced in this study.

## Additional Points

*Highlights*. (1) *Stenotrophomonas* D0Y85_RS06240 is related to cell envelope integrity proteins. (2) D0Y85_RS06240 mediates multiantibiotic resistance. (3) D0Y85_RS06240 is inhibited by efflux pump inhibitor. (4) D0Y85_RS06240 binds to colistin, tetracycline, and cefixime.
